# β2-Adrenergic receptor agonist enhances the bystander effect of HSV-TK/GCV gene therapy in glioblastoma multiforme via upregulation of connexin 43 expression

**DOI:** 10.1016/j.omto.2022.05.010

**Published:** 2022-06-06

**Authors:** Saereh Hosseindoost, Seyed Mojtaba Mousavi, Ahmad Reza Dehpour, Seyed Amirhossein Javadi, Babak Arjmand, Ali Fallah, Mahmoudreza Hadjighassem

**Affiliations:** 1 Pain Research Center, Neuroscience Institute, Tehran University of Medical Sciences, Tehran, Iran; 2 Department of Neuroscience and Addiction Studies, School of Advanced Technologies in Medicine, Tehran University of Medical Sciences, Italia St, Tehran, Iran; 3 Department of Pharmacology, School of Medicine, Tehran University of Medical Sciences, Tehran, Iran; 4 Experimental Medicine Research Center, Tehran University of Medical Sciences, Tehran, Iran; 5 Brain and Spinal Cord Injury Research Center, Neuroscience Institute, Tehran University of Medical Sciences, Keshavarz Blvd, Dr. Gharib St, Tehran, Iran; 6 Neurosurgery Department, Imam Khomeini Hospital Complex, TUMS, Tehran, Iran; 7 Cell Therapy and Regenerative Medicine Research Center, Endocrinology and Metabolism Molecular-Cellular Sciences Institute, Tehran University of Medical Sciences, Tehran, Iran; 8 Metabolomics and Genomics Research Center, Endocrinology and Metabolism Molecular-Cellular Sciences Institute, Tehran University of Medical Sciences, Tehran, Iran; 9 Systems and Synthetic Biology Group, Mede Bioeconomy Company, Tehran, Iran; 10 Space Medicine B.V., Rotterdam, the Netherlands

**Keywords:** β2-adrenergic receptor, connexin 43, glioblastoma multiforme, olfactory ensheathing cells, gene therapy, bystander effect

## Abstract

Glioblastoma multiforme (GBM) is the most invasive form of primary brain astrocytoma. Gene therapy using the herpes simplex virus thymidine kinase/ganciclovir (HSV-TK/GCV) is a new strategy for GBM treatment. As the connexin 43 (Cx43) levels are downregulated in GBM cells, it seems that the upregulation of Cx43 could improve the efficacy of the gene therapy. This study aims to evaluate the effect of clenbuterol hydrochloride (Cln) as a β2-adrenergic receptor agonist on HSV-TK/GCV gene therapy efficacy in human GBM cells using olfactory ensheathing cells (OECs) as vectors. The lentivirus containing the thymidine kinase gene was transduced to OECs and the effective dose of GCV on cells was measured by MTT assay. We found that Cln upregulated Cx43 expression in human GBM cells and OECs and promoted the cytotoxic effect of GCV on the co-culture cells. Western blot results showed that Cln increased the cleaved caspase-3 expression and the Bax/Bcl2 ratio in the co-culture of GBM cells and OEC-TK. Also, the flow cytometry results revealed that Cln increased apoptosis in the co-culture of GBM cells and OEC-TK cells. This study showed that Cln via upregulation of Cx43 expression could enhance the bystander effect of HSVTK-GCV gene therapy in human GBM cells.

## Graphical abstract

**Figure:**
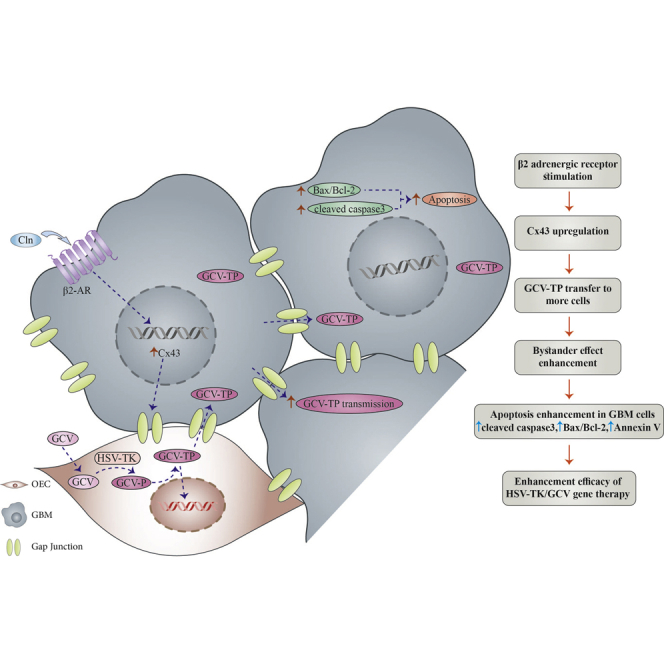

## Teaser

Cln as a β2-AR agonist via the upregulation of Cx43 expression can increase the permeability of GCV-TP to GBM cells and, through the enhancement of the bystander effect between the cells, could increase the efficacy of gene therapy in these tumors.

## Introduction

Glioblastoma multiforme (GBM), categorized as grade IV astrocytoma, is the most prevalent and invasive type of primary brain tumor in adults. GBM accounts for approximately 80% of malignant gliomas.^1^^,^^2^ The current standard treatments for GBM patients include the combination of maximal surgical resection, radiotherapy, and chemotherapy.^2^^,^^3^ Despite these treatments, clinical outcomes are not successful and the median overall survival of patients is about 10–16 months in most cases.^4^, ^5^, ^6^ Therefore, researchers are investigating strategies for effective treatment approaches in GBM patients. Currently, suicide gene therapy using the herpes simplex virus thymidine kinase/ganciclovir (HSV-TK/GCV) is a promising strategy for GBM treatment.^7^^,^^8^ In this approach, prodrug GCV is phosphorylated by the HSV-TK to the GCV monophosphate, which is then converted to cytotoxic GCV triphosphate (GCV-TP) using endogenous kinases. The GCV-TP is incorporated into the DNA strand during replication and leads to apoptotic cell death.^9^, ^10^, ^11^ Moreover, the GCV-TP can be transmitted to adjacent cells via gap junctional intercellular communication (GJIC) and induces apoptosis in the neighboring tumor cells that do not express the TK gene. The bystander effect refers to the destruction of tumor cells that do not contain the TK gene, but indirectly undergo apoptosis due to the transmission of toxic metabolites.^12^, ^13^, ^14^ Despite remarkable outcomes of GBM gene therapy in experimental research, the clinical trials results were not very successful.^15^^,^^16^ It seems that a major limitation of glioma gene therapy is reduced intercellular communication and insufficient cytotoxic metabolite transmission to whole tumor cells. Previous studies showed that tumor cells undergo some molecular changes that may affect the response to treatments. Several studies reported lower GJIC in astrocytic tumors and glioma samples.^17^, ^18^, ^19^ Sufficient intercellular communication between tumor cells can effectively transfer cytotoxic drugs to whole tumor tissue and destroys all tumor cells.^19^, ^20^, ^21^ GBM cells, especially astrocytes, are connected through gap junctions. Gap junction channels are comprised of connexins and the most predominant connexin in the human brain is connexin 43 (Cx43).^22^^,^^23^ Some studies revealed that the level of Cx43 expression is inversely related to glioma grade.^24^^,^^25^ Therefore, the insufficient response to gene therapy may be due to this reduction in communication between tumor cells. Thus, strategies that enhance GJIC levels on both tumor cells and vectors may generally improve gene therapy efficiency. In gene therapy experiments, various vectors, such as viruses, stem cells, and, lately, olfactory ensheathing cells (OECs), are used to transfer cytotoxic metabolites to tumor cells.^26^, ^27^, ^28^, ^29^

OECs are glial-like cells that are mainly located in the olfactory bulbs and olfactory mucosa. They express glial markers, such as S100β, GFAP, and p75NTR.^30^ Some of the advantages of OECs, such as accessible source and high migration potential without any risk of tumorigenesis, have made them appropriate candidates for gene therapy studies.^25^^,^^27^^,^^31^^,^^32^ β2-Adrenergic receptor (β2-AR) signaling is an important pathway to the regulation of Cx43 levels.^33^^,^^34^ Our previous study indicated that clenbuterol hydrochloride (Cln), a selective β2-AR agonist, enhanced the Cx43 expression levels in human GBM cells and OECs.^35^ In this study, we hypothesize that the stimulation of β2-AR via the upregulation of Cx43 expression strengthens bystander effect and improves the gene therapy in GBM cells ultimately. This study aimed to evaluate the effect of Cln on the bystander effect between OEC-TK and GBM cells in HSV-TK/GCV suicide gene therapy using OECs as vectors.

## Results

### Isolation and characterization of human GBM-derived astrocytes

**Figure 1:**
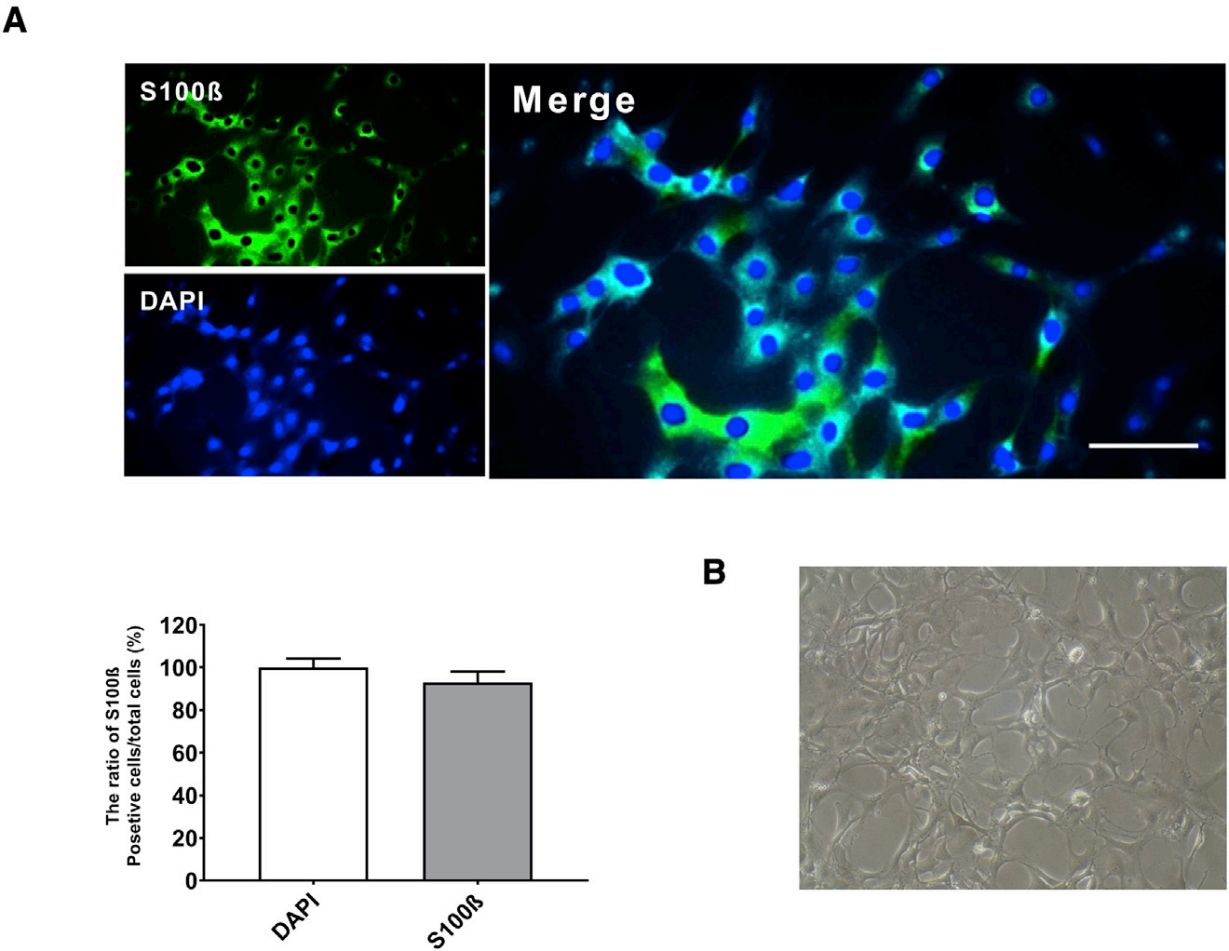
Morphology of human GBM-derived astrocytes Immunofluorescence staining was performed to detect the expression of the S100-β antigen in astrocyte cells. (A) Approximately 92% of cells expressed S100-β antigen. Scale bar, 50 μm. (B) The cells presented star-like morphology with branching processes.

In this research, we first isolated and purified the astrocytes from GBM tissues, and this purification was verified using phase-contrast microscopy and immunofluorescence staining. The immunofluorescence technique showed that primary cultured astrocyte cells exhibited positive S100-β staining. Our results showed that more than 90% of cells expressed S100-β antigen as an astrocyte marker (Figure 1A). Furthermore, primary astrocytes typically showed a polygonal shape and star-like morphology with complex branched processes (Figure 1B).

### Evaluation the effect of the β2-AR agonist on Cx43 expression in human GBM-derived astrocytes and human OECs

**Figure 2:**
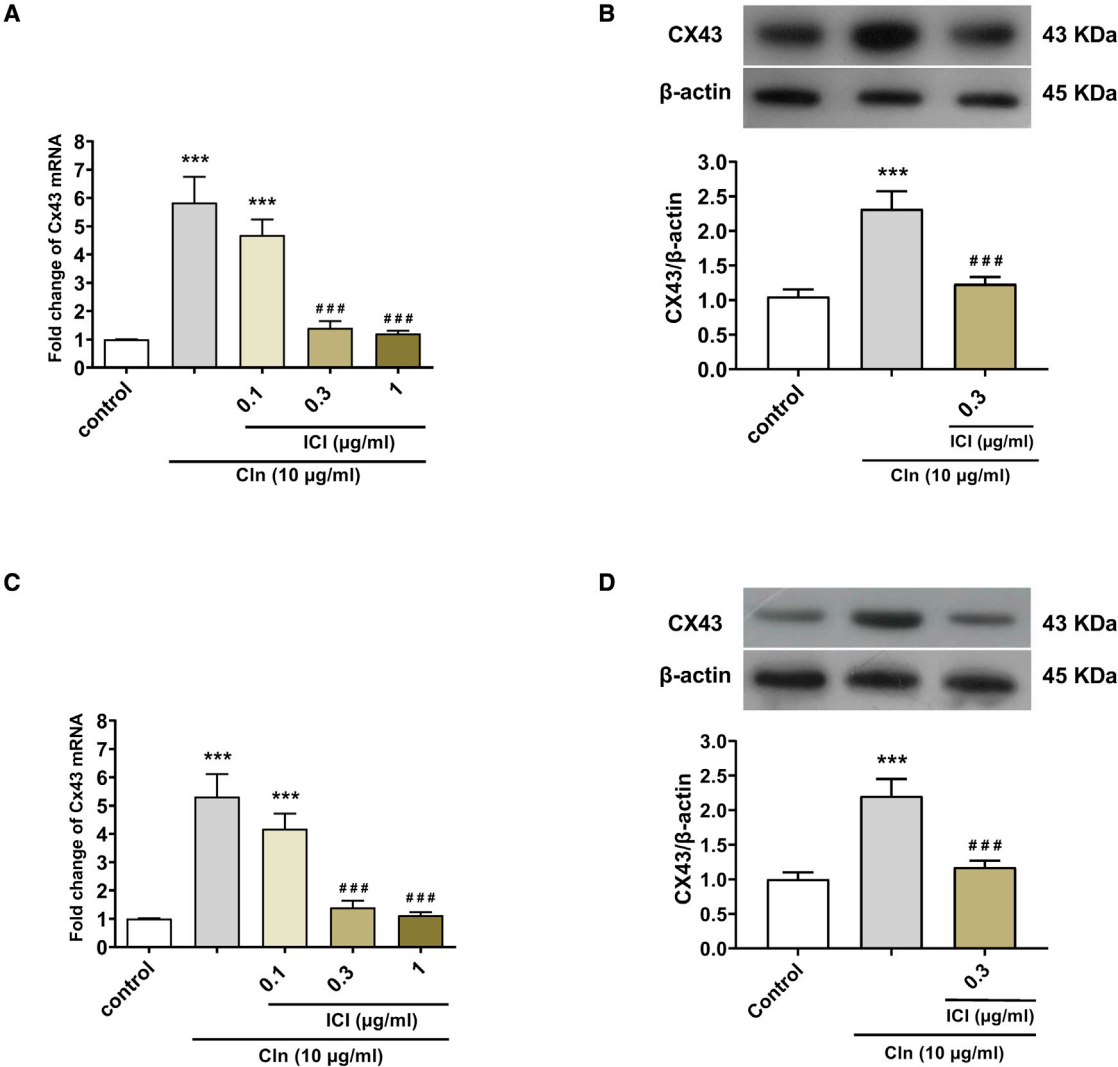
Cln as a β2-AR agonist upregulated Cx43 expression levels in human GBM cells and human OECs (A) Real-time PCR analysis for Cx43 expression in GBM cells. (B) Western blot analysis for Cx43 levels in GBM cells. (C) Real-time PCR results for Cx43 expression in OECs. (D) Western blot results for Cx43 levels in OECs. The cells were treated with Cln and ICI for 24 h according to the grouping. Data are shown as means ± SD (n = 3). ∗∗∗p < 0.001 versus control, ###p < 0.001 versus Cln. p values were assessed with one-way ANOVA followed by Tukey’s post hoc test.

To investigate the effect of the β2-AR agonist on Cx43 expression in GBM cells and OECs, we evaluate the effect of Cln on Cx43 levels using real-time PCR and the western blot technique. The cells were incubated with Cln at a concentration of 10 μg/mL and ICI at concentrations of 0.1, 0.3, and 1 μg/mL according to the groups for 24 h. Real-time PCR results showed that the mRNA levels of Cx43 dramatically elevated after exposure to Cln at a concentration of 10 μg/mL in GBM cells. To confirm the upregulation of Cx43 levels mediated with β2-AR, the GBM cells were pretreated with different concentrations of ICI for 45 min. Our results revealed that ICI at 0.3 and 1 μg/mL concentrations inhibited the Cln effect on Cx43 expression, although the dose of 0.1 μg/mL did not suppress its effect. Based on these data, we selected 0.3 μg/mL ICI for assessing the inhibitory effect on β2-AR (Figure 2A). To evaluate the effect of Cln on protein levels of Cx43, we measured the expression of Cx43 through western blot analysis. These results, similar to our previous findings,^35^ show that Cln (10 μg/mL) could significantly increase the protein levels of Cx43 in GBM cells. Also, ICI at a concentration of 0.3 μg/mL blocked the Cln effect on Cx43 protein expression (Figure 2B). These findings demonstrated that the Cln effect on the expression of Cx43 might be mediated with β2-AR.

Moreover, our real-time PCR results revealed that Cln at a concentration of 10 μg/mL could significantly increase the mRNA levels of Cx43 in OECs. To investigate whether the Cln effect on Cx43 expression was mediated through β2-AR, the OECs were pretreated with ICI. The data showed that ICI at 0.3 and 1 μg/mL concentrations prevented the Cln effect on Cx43 upregulation. Nevertheless, the concentration of 0.1 μg/mL ICI did not prevent this effect (Figure 2C). Furthermore, similar to our previous study, western blot analysis demonstrated that the protein levels of Cx43 significantly upregulated after treatment with Cln at a concentration of 10 μg/mL and ICI (0.3 μg/mL) repressed its effect (Figure 2D). These findings indicated that Cln could upregulate Cx43 expression in OECs, and its effect probably was mediated through β2-AR.

### Expression of TK gene in OECs

**Figure 3:**
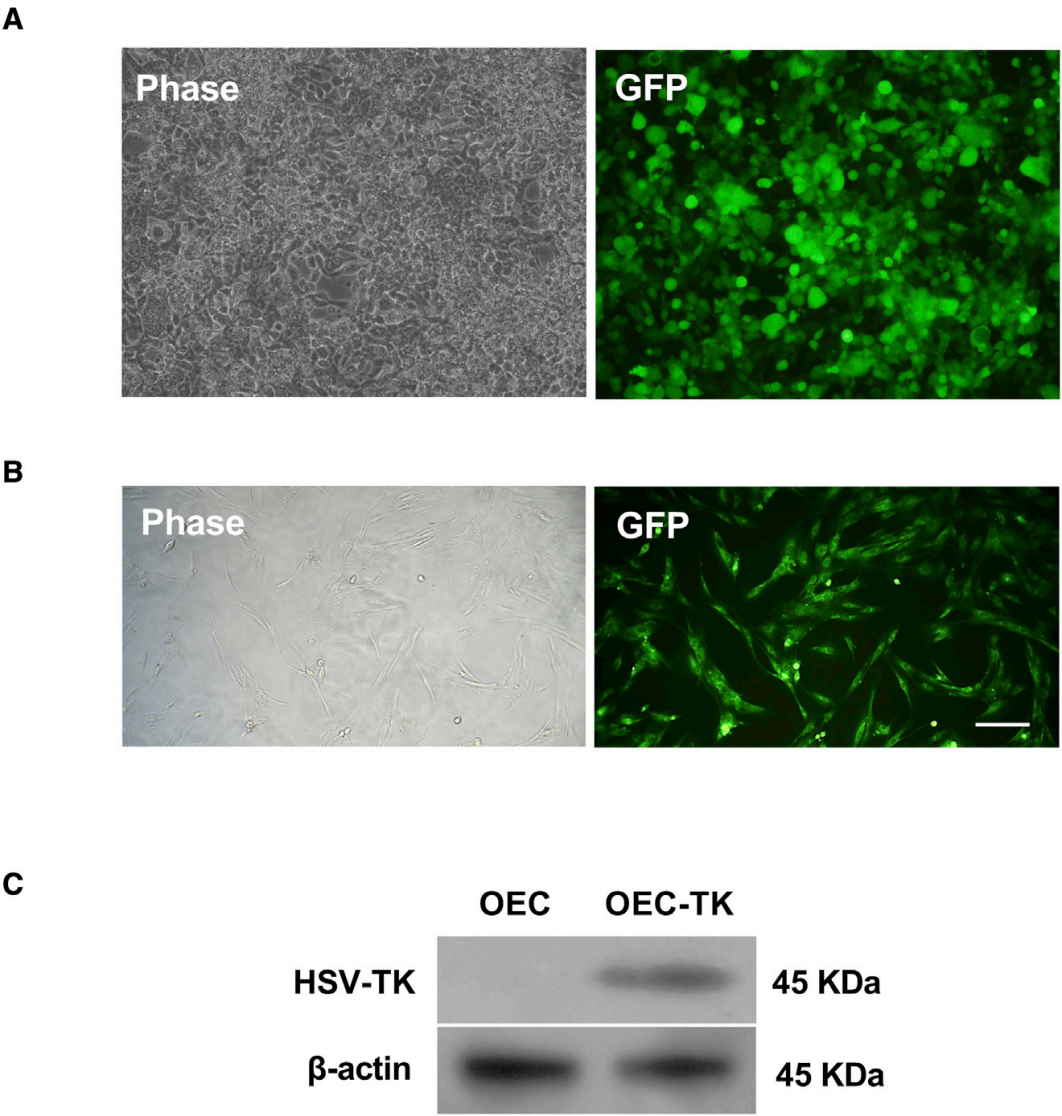
Confirmation of HSV-TK transduction in OECs (A) GFP expression by HEK293 cells was detected with fluorescence microscopy. (B) Transduction efficiency was detected by GFP signal under a fluorescence microscope after 72 h post-transduction. Scale bar, 50 μm. (C) Western blot results show that the TK protein band was only detected in OEC-TK groups.

The GFP reporter gene expression is an index of transfection efficiency in lentiviral vectors. As shown in Figure 3A, GFP expression by HEK293 cells demonstrated that lentiviral vectors were successfully transfected and expressed in the HEK293 cells (Figure 3A). This research transduces the lentiviral vector containing HSV-TK/GFP in OECs. To determine whether the HSV-TK/GFP successfully transduced into OECs and TK expressed in cells, the GFP signal was detected using fluorescence microscope, and the TK expression was evaluated using western blot techniques. GFP reporter gene is an index of successfully HSV-TK/GFP expression and transduction. Under the fluorescence microscope, a robust green fluorescence signal was observed in transduced OECs (OEC-TK) after 72 h of transduction (Figure 3B). Moreover, our western blot analysis showed that TK protein was strongly expressed in OEC-TK but not in OEC groups (Figure 3C). These findings suggest that HSV-TK was stably expressed in OECs.

### Evaluation the OEC-TK sensitivity to GCV and bystander effect between OEC-TK and GBM-derived astrocytes

**Figure 4:**
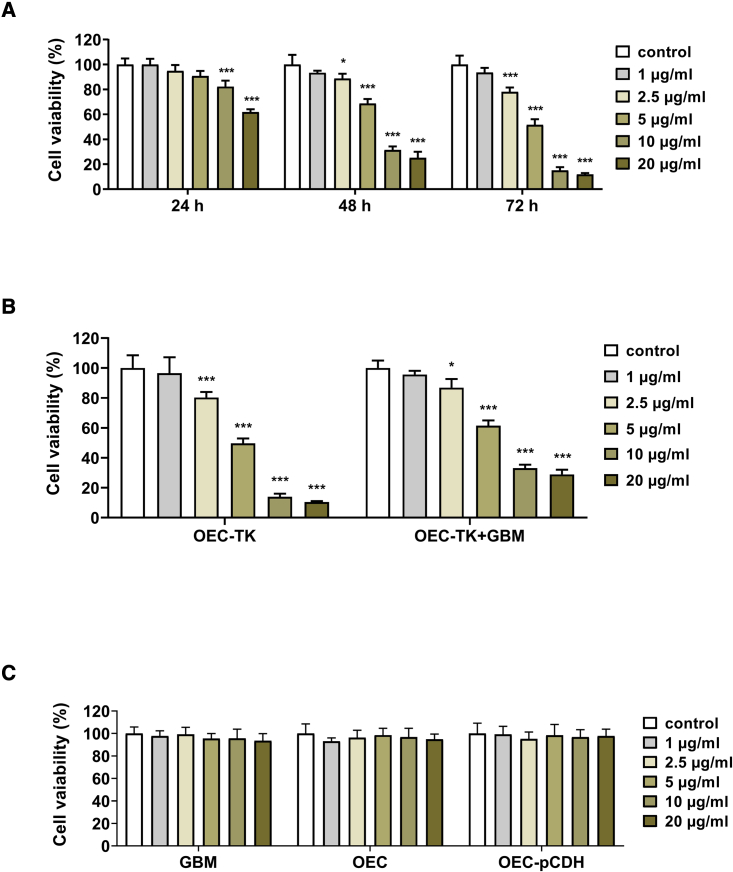
Determination of OEC-TK sensitivity to GCV and bystander effect between OEC-TK and GBM cells (A) The OEC-TK were incubated with different concentrations of GCV several times. The cell viability was measured by MTT assay. (B) The bystander effect between OEC-TK and GBM cells was evaluated. The OEC-TK were co-cultured with GBM cells in a ratio of 1:1 and the cells were treated with various concentrations of GCV for 72 h. (C) The GBM cells, OECs, and OEC-pCDH were exposed to different concentrations of GCV for 72 h and the viability of cells was evaluated. Data are shown as means ± SD (n = 3). ∗p < 0.05, ∗∗∗p < 0.001 versus control. p values were measured with one-way ANOVA and two-way ANOVA followed by Tukey’s post hoc test.

This experiment investigated the effective concentration and time of GCV treatment on OEC-TK and the appropriate dose of GCV treatment for bystander effect between the OEC-TK and GBM cells. The suitable concentration and treatment time of GCV on OEC-TK was determined using an MTT (3-(4, 5-dimethylthiazol-2-yl)-2,5-diphenyltetrazolium bromide) assay. OEC-TK were treated with increasing concentrations of GCV (1–20 μg/mL) for 24, 48, and 72 h. According to our results, the GCV exerted a dose- and time-dependent cytotoxicity on OEC-TK. The MTT results indicated that the concentrations of 10 and 20 μg/mL of GCV during a treatment duration of 24 h and the concentrations of 2.5–20 μg/mL for 48 and 72 h significantly decreased cell viability of OEC-TK. Based on these findings, the treatment duration of 72 h was chosen for the following bystander effect experiments (Figure 4A).

To evaluate the bystander effect between the OEC-TK and GBM cells, OEC-TK were co-cultured with GBM cells at a ratio of 1:1 and were incubated with different concentrations of GCV (2.5–20 μg/mL) for 72 h. Similar to the previous results, when the OEC-TK were alone exposed to the GCV at concentrations of 2.5–20 μg/mL, the viability of the cells was dramatically reduced. Moreover, the bystander killing effect was observed in OEC-TK and GBM cells' co-culture systems. When the mix of these cells was treated with 2.5–20 μg/mL of GCV, the viability of cells was significantly reduced in a concentration-dependent manner (Figure 4B). Since the GCV at a concentration of 2.5 μg/mL significantly reduced the viability of cells in the co-culture system, we selected this concentration for the following experiments.

Next, the effect of GCV on the cells without the TK gene was investigated. In contrast to the OEC-TK, no cytotoxic effect was observed in GBM cells, OECs, and OEC-pCDH (OECs containing the empty vector), which were treated with various concentrations of GCV at 72 h. These results revealed that the viability of cells that did not contain the TK gene was not decreased on exposure to GCV (Figure 4C). Therefore, the presence of the TK protein is essential for the conversion of GCV to a cytotoxic phosphorylated form, and without the TK gene, cells will not be destroyed.

### Evaluation the effect of β2-AR agonist on the bystander effect between OEC-TK and GBM-derived astrocytes

**Figure 5:**
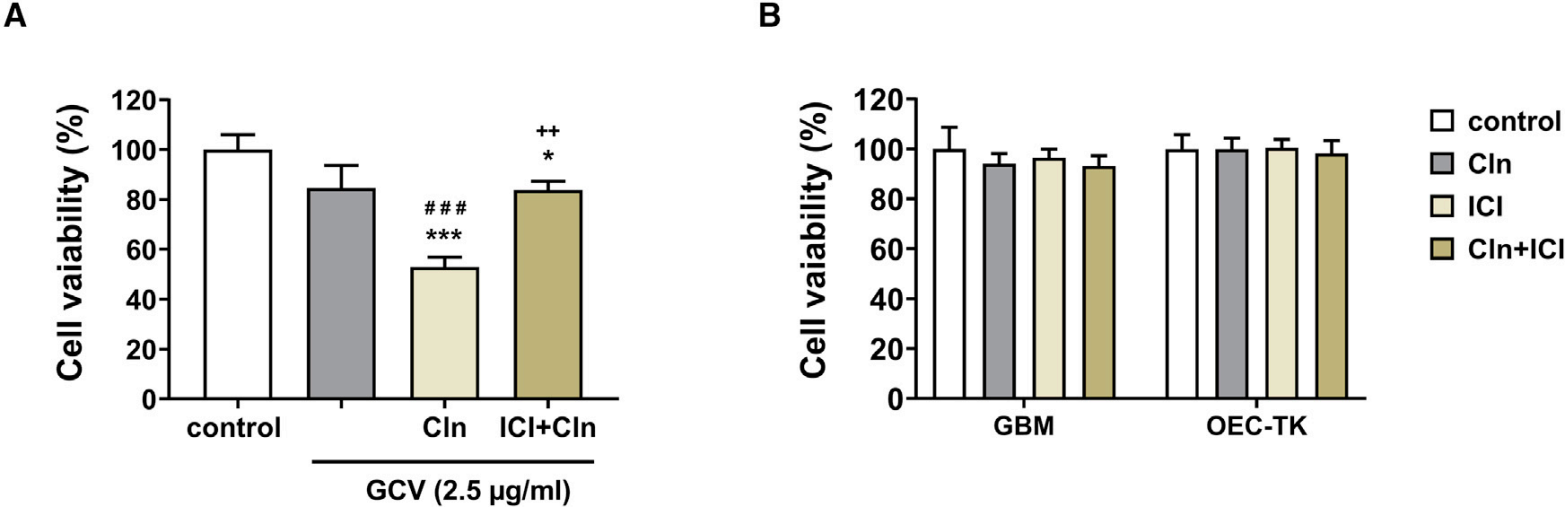
Cln enhance the bystander effect between OEC-TK and GBM cells (A) The OEC-TK and GBM cells were mixed at a ratio of 1:1 and treated with GCV (2.5 μg/mL), Cln (10 μg/mL), and ICI (0.3 μg/mL) for 72 h according to the grouping. The viability of cells was evaluated with MTT assay. (B) The GBM cells and OEC-TK were exposed to Cln (10 μg/mL) and ICI (0.3 μg/mL) for 4 days for 72 h and the cell viability was measured. Data are presented as means ± SD (n = 3). ∗p < 0.05, ∗∗∗p < 0.001 versus control. ###p < 0.001 versus GCV. ++p < 0.01 versus Cln + GCV. p values were assessed with one-way ANOVA followed by Tukey’s post hoc test.

The previous results demonstrated that Cln as a β2-AR agonist could upregulate Cx43 expression in GBM cells and OECs.^35^ Since the levels of the Cx43 and GJIC play a substantial role in mediating the bystander effect, in this section we evaluate the effect of Cln on the bystander effect between OEC-TK and GBM cells and the efficacy of HSV-TK/GCV suicide gene therapy of GBM cells. The OEC-TK and GBM cells were co-cultured at a ratio of 1:1 and incubated with GCV (2.5 μg/mL), Cln (10 μg/mL), and ICI (0.3 μg/mL) according to the grouping. The MTT results indicated that, after GCV treatment alone, only a small suppression of viability was observed in the cells. Cell survival in the GCV treatment group was not significantly different from the untreated group. Indeed, Cln together with GCV significantly decreased the viability of cells compared with GCV treatment alone. Treatment with Cln was able to augment the OEC-TK-mediated cell killing. In other words, Cln elevated the bystander effect of OEC-TK on the GBM cells. On the other hand, when the cells were pretreated with ICI, the Cln effect on the viability of the cells was significantly reversed. Treatment with ICI significantly abrogated the Cln effect on the bystander killing effect (Figure 5A). These findings suggest that Cln can increase the cell death of GBM cells by enhancing the GJIC and thus can improve the efficacy of HSV-TK gene therapy, and these effects may be mediated with β2-AR.

Furthermore, to determine the effect of Cln (10 μg/mL) and ICI (0.3 μg/mL) alone and also their combination on the viability of GBM cells and OEC-TK, cells were exposed to these drugs for 4 days, and the cell viability was evaluated by MTT assay. Our results revealed that Cln and ICI alone and their combination have no cytotoxic effect on OEC-TK and GBM cells. The viability of cells did not show significant differences compared with the control group (Figure 5B).

### Evaluation the effect of β2-AR agonist on apoptosis induction of OEC-TK/GCV suicide gene therapy

Finally, we studied the effect of Cln on the apoptosis induction of OEC-TK. To evaluate apoptosis, the expressions of cleaved caspase-3 and Bax (as the pro-apoptotic proteins) and Bcl2 (as an anti-apoptotic protein) were measured using western blot, and the percentage of apoptotic cells was assessed by flow cytometry. The co-cultures of OEC-TK and GBM cells were exposed to GCV (2.5 μg/mL), Cln (10 μg/mL), and ICI (0.3 μg/mL) according to the groups.

**Figure 6:**
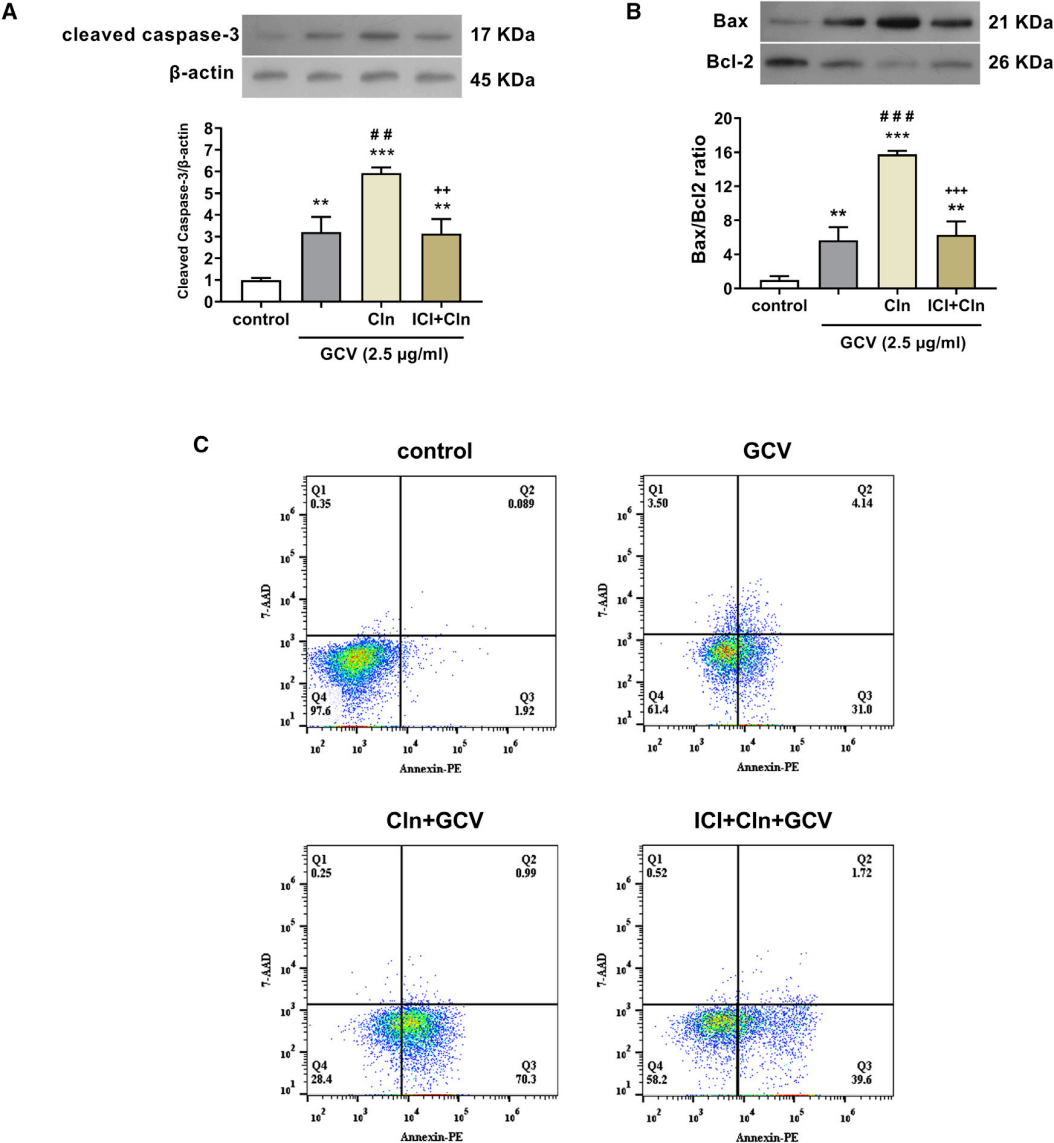
Cln enhances the apoptosis induction of OEC-TK/GCV gene therapy of GBM cells (A and B) The OEC-TK and GBM cells were co-culture at a ratio of 1:1 and treated with GCV (2.5 μg/mL), Cln (10 μg/mL), and ICI (0.3 μg/mL) for 72 h according to the grouping. The expression levels of cleaved caspase-3, Bax, and Bcl2 were assessed by western blot. (C) The percentage of apoptotic cells was measured by flow cytometry. Data are presented as means ± SD (n = 3). ∗∗p < 0.01, ∗∗∗p < 0.001 compared with control. ##p < 0.01, ###p < 0.001 compared with GCV. ++p < 0.01, +++p < 0.001 compared with Cln + GCV. p values were measured by one-way ANOVA followed by Tukey’s post hoc test.

Our western blot analysis indicated that GCV treatment alone could significantly upregulate the cleaved caspase-3 levels than the control group. When the cells were exposed to the combination of Cln and GCV, the expression of the cleaved caspase-3 protein was dramatically increased compared with the GCV treatment alone. On the other hand, ICI treatment suppressed the Cln effect on the cleaved caspase-3 expression (Figure 6A). Furthermore, our results showed that treatment with GCV significantly enhanced the Bax expression levels and reduced the Bcl-2 levels versus the control. Indeed, the ratio of Bax/Bcl-2 was enhanced in the GCV group versus in untreated cells. When the cells were treated by Cln simultaneously with GCV, the ratio of Bax/Bcl-2 drastically elevated compared with the GCV treatment alone. Moreover, ICI treatment prevented the Cln effect on the expression of these proteins (Figure 6B).

Furthermore, flow cytometry with 7-AAD stain was carried out to analyze the co-culture apoptosis of the OEC-TK and GBM cells. The results showed that GCV treatment alone induces apoptosis in approximately 35% of cells. The percentage of apoptotic cells in the GCV group was significantly increased compared with the untreated cells. Cln treatment in combination with GCV enhanced the percentage of apoptotic cells by up to 70%, and the apoptosis was significantly elevated compared with the GCV treatment group. In other words, Cln could remarkably enhance the OEC-TK/GCV-induced apoptosis. On the other hand, the Cln effect on apoptosis induction was abrogated by ICI treatment (Figure 6C).

## Discussion

**Figure 7:**
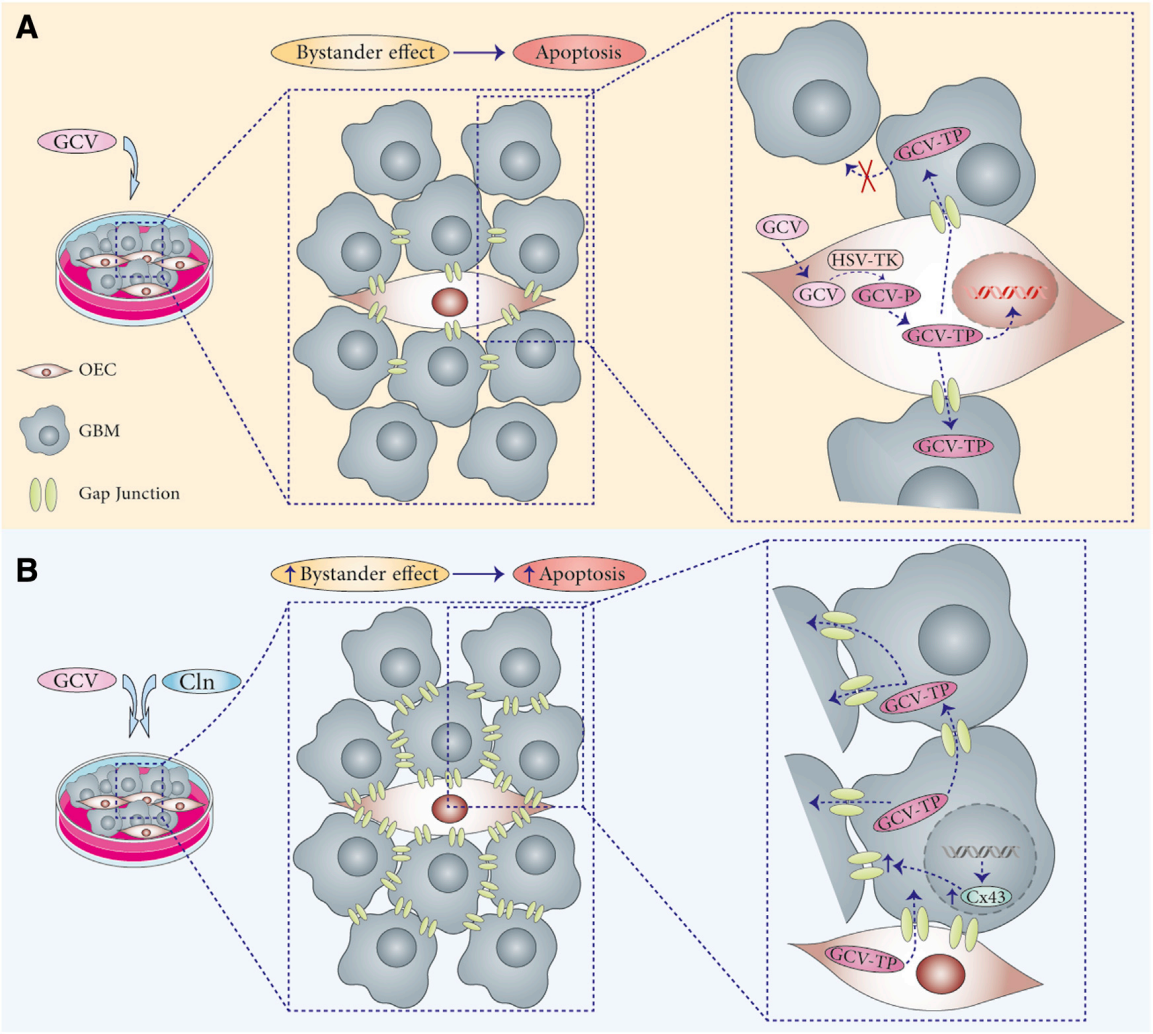
Schematic diagram of bystander effect between OEC-TK and GBM cells and the effect of Cln on this phenomenon (A) The cells are connected by gap junction channels. The HSV-TK gene only expresses in one cell (OEC-TK). Treatment of the cell population by non-toxic GCV leads to monophosphorylation of GCV (GCV-P) with the HSV-TK enzyme in the OEC-TK. Endogenous kinases within the cells convert GCV-P to toxic product (GCV-TP). Gap junctions between cells facilitate the transmission of GCV-TP to neighboring non-transduced GBM cells. GCV-TP is incorporated into replicating DNA in OEC-TK and GBM cells, resulting in the apoptosis of cells. The death of non-transduced GBM cells due to the transfer of a cytotoxic metabolite (GCV-TP) is known as the bystander effect. (B) Pretreatment of cell population with Cln leads to upregulation of Cx43 expression and enhancement of gap junctions between cells. Following the enhancement of GJIC, GCV-TP can be transferred to more GBM cells and induces apoptosis in them, consequently leading to promotion of the bystander effect.

GBM is the most aggressive and frequently occurring form of primary brain tumors in adults.^5^ HSV-TK/GCV suicide gene therapy is an encouraging approach for GBM treatment. The effective transfer of GCV-TP from vectors to tumor cells is an important factor in this approach. GCV-TP transmission depends on the GJIC between cells. More GJIC results in the transmission of GCV-TP to the deeper layer of tumors. Thus, the upregulation of Cx43 expression gates accessibility of cytotoxic metabolite to deeper layers of GBM (Figure 7). In our previous study, we showed that β2-AR stimulation enhanced the Cx43 expression levels in human GBM cells and OECs.^35^ In this study, we tried to demonstrate that targeting GBM tumor cells by OEC-HSV-TK/GCV in the presence of a β2-AR agonist enhanced the apoptotic effect of our construct.

The GBM tissues are composed of a complex heterogeneous nature, and astrocytes, oligodendrocytes, fibroblasts, cancer stem cells, and innate and adaptive immune cells were present in the GBM stroma.^36^ Astrocytes are the most important cells of GBM tissue, which highly express the S100-β calcium-binding protein. They have a high proliferative capacity and play an important role in the pathophysiology of GBM,^37^, ^38^, ^39^ so we purified the astrocytes from GBM tissue in our research. Several studies reported that Cx43 expression was downregulated in high-grade glioma tumors,^18^^,^^40^^,^^41^ and previous studies showed that stimulation of the cAMP pathway enhanced the Cx43 expression in glioma cells.^42^^,^^43^ In our experiment, Cln (as a β2-AR agonist) was used for the upregulation of Cx43 levels in GBM cells and OECs. Our finding revealed that Cln upregulates the mRNA and protein levels of Cx43 expression in GBM cells and OECs. To confirm the role of β2-AR on the upregulation of Cx43 expression, ICI was used as a β2-AR antagonist to block the receptor. Pretreatment with ICI suppressed the Cln effect on Cx43 expression in both GBM cells and OECs. These results are consistent with our previous study, which revealed that Cln increased the Cx43 protein levels in the primary astrocytes and OECs.^35^ Consistent with our results, other studies have shown that the signaling pathways induced by β2-AR stimulation lead to the upregulation of Cx43 levels in various cells.^33^^,^^34^^,^^44^, ^45^, ^46^ Since Cx43 is the major structure of GJIC, which is an important factor in GCV-TP transmission, we evaluated the effect of Cln on apoptosis induction of HSV-TK/GCV gene therapy in GBM cells. The question was addressed as to whether increased Cx43 expression leads to the induction of apoptosis by HSV-TK/GCV in more cells and thus increases the efficiency of gene therapy.

Considering the role of β2-AR in the upregulation of Cx43 expression, our study showed that Cln could enhance the bystander effect between the OEC-TK and GBM cells in the co-culture system and ICI reverse its effect. It seems that Cln can improve the bystander effect by augmenting the expression of Cx43 and consequently transfer GCV-TP to more cells through the β2-AR. Several gene therapy studies demonstrated that the upregulation of Cx43 expression plays an essential role in enhancing the bystander effect in various cells.^47^, ^48^, ^49^

Our results and previous studies have shown that the enhancement of the bystander effect is associated with elevated cell death. Since apoptosis is the main mechanism of cell death induced by HSV-TK/GCV,^50^^,^^51^ to investigate the responsible pathway for cell death in our experiment, we evaluated the effect of Cln on apoptosis in the co-culture of GBM cells and OEC-TK cells. Apoptosis is a natural physiological process to remove damaged cells and apoptosis induction is an effective strategy in the treatment of cancers.^52^^,^^53^ The caspases and Bcl-2 family are the most important proteins involved in tumor cell apoptosis. Two main members of the Bcl-2 family include Bax (pro-apoptotic) and Bcl-2 (anti-apoptotic).^54^ The balance between Bax and Bcl-2 proteins can be seen by evaluating the sensitivity of tumor cells to GCV.^55^ According to some research, the expression of Bcl-2 in glioma tumors is elevated compared with normal astrocytes.^56^ In addition, upregulation of Bax expression induces cell death of tumor cells.^55^^,^^57^

Our finding indicated that GCV could significantly upregulate the pro-apoptotic protein levels (cleaved caspase-3 and Bax) and downregulate the anti-apoptotic protein expression (Bcl-2) in HSV-TK/GCV gene therapy. Indeed, Cln treatment could reinforce the GCV effect on these protein expression levels. Consistence with our results, several studies revealed that HSV-TK/GCV enhances the pro-apoptotic protein and reduces the anti-apoptotic protein levels in other cells.^51^^,^^55^^,^^58^^,^^59^ Based on these findings, it can be concluded that GCV-TP, through incorporation into the DNA strands, triggers the intracellular apoptotic pathway, alters the expression of Bax and Bcl-2 proteins, and activates caspase-3. Moreover, by increasing the Cx43 levels Cln promotes the effect of GCV-TP on apoptosis induction on cells. In line with these results, our flow cytometry data showed that GCV increased the rate of apoptosis compared with the control group. Moreover, treatment with Cln enhanced apoptosis induction compared with the GCV group. In other words, OEC-TK/GCV-induced apoptosis was remarkably enhanced in the presence of Cln. Similar to our findings, another report indicated that the upregulation of Cx43 and the subsequent enhancement of GJIC could increase Annexin V and apoptosis induction of HSV-TK/GCV gene therapy in melanoma cells.^60^

Since this study, as a preliminary experiment, has been performed on primary cells isolated from GBM tumors, its results can be relatively generalized to whole human GBM tissue. However, for more accurate conclusions, additional β2-AR agonists and antagonists should be studied to examine the effect of β2-AR on Cx43 expression and HSV-TK/GCV gene therapy. Since, in the *in vitro* studies, the effects of the microenvironment of the tumors are not examined, and the roles of the immune system, angiogenesis, and other cells of the tumor microenvironment are not considered, it is necessary to investigate the effects of this combinational therapy in animal and pre-clinical studies.

## Conclusion

According to the obtained results, it can be concluded that the β2-AR agonist, via the upregulation of Cx43 expression, can increase the permeability of cytotoxic metabolites, such as GCV-TP, to GBM cells and thus, through the enhancement of the bystander effect between the cells, could increase the efficacy of HSV-TK/GCV gene therapy in these tumors.

## Materials and methods

### Reagents

The drugs, including Cln and GCV, were purchased from Sigma-Aldrich (St. Louis, MO, USA), and ICI 118551 hydrochloride (ICI) was purchased from Tocris Bioscience (Bristol, UK). The antibodies, including rabbit monoclonal anti-S100-β, rabbit polyclonal anti-Cx43, goat anti-rabbit IgG H&L (HRP), and goat polyclonal anti-rabbit IgG H&L (Alexa Fluor 488) were bought from Abcam (Cambridge, MA, USA). The rabbit polyclonal primary antibody against cleaved caspase-3 was purchased from Cell Signaling Technology (Beverly, MA, USA), anti-Bax, anti-Bcl-2, and anti-TK were bought from Santa Cruz Biotechnology (Santa Cruz, CA, USA). The rabbit polyclonal anti-β-actin was obtained from PADZA (Tehran, Iran). DMEM/F12, fetal bovine serum (FBS), antibiotic/antimycotic, trypsin-EDTA, and MTT were purchased from Gibco Life Technologies (Carlsbad, CA, USA). The Annexin V/7-AAD detection kit, TRIzol reagent, and the reverse transcription kit were procured from BioLegend (San Diego, CA, USA), Thermo Fisher Scientific (Waltham, MA, USA), and Takara (Shiga, Japan), respectively. The enhanced chemiluminescence (ECL) kit was bought from Amersham Biosciences (Freiburg, Germany).

### Human GBM tumor culture

GBM tissue samples were collected from patients who underwent surgical resection at the Department of Neurosurgery, Imam Khomeini Hospital, Tehran, Iran. The histopathological analysis showed that the tissue samples have the diagnostic criteria of GBM. Fresh tumor specimens, immediately after resection, were placed into cold PBS containing 5% antibiotic/antimycotic. The samples were washed in cold PBS and then dissociated into small pieces with a scalpel. For enzymatic dissociation, tumor tissues were trypsinized using 0.25% trypsin-EDTA and incubated at 37°C for 15 min. Trypsin inactivation was carried out using a medium containing 10% FBS, and then these suspensions were centrifuged for 5 min at 1,200 × *g*. The cells were finally suspended and cultured into DMEM/F12 medium supplemented with 2% FBS, 1% antibiotic/antimycotic, and incubated at 37°C in 5% CO_2_. FBS concentration was gradually elevated to 10% during medium change for 2 weeks.

### Human OEC culture

OECs were isolated and characterized as in the previous study.^27^ The OECs were cultured in DMEM/F12 medium containing 10% FBS and 1% antibiotic/antimycotic. The cells were cultured at a density of 10,000 cells/cm^2^ and maintained in a 5% CO_2_ atmosphere at 37°C.

### Immunofluorescence

The cells (at a density of 5 × 104 per well) were cultured in a 24-well tissue plate. After 24 h, the wells were washed two times with PBS and fixed in 4% paraformaldehyde for 20 min at room temperature. Next, the cells were washed three times with PBS and permeabilized using 0.2% Triton X-100 for 15 min. The nonspecific binding sites were blocked by 3% bovine serum albumin for 1 h. The cells were then incubated with primary antibodies against S100-β (1:200) overnight at 4°C. After washing three times with PBS, The cells were incubated with goat anti-rabbit IgG H&L (Alexa Fluor 488) for 2 h in the dark at room temperature. Finally, the nuclei were stained with DAPI for 10 min at room temperature and washed with PBS. An inverted fluorescence microscope was used for imaging.

### Study design and treatments

This study was performed in two steps. In the first step, we evaluated the effect of Cln on the Cx43 expression levels in GBM cells and OECs. The cells were treated with Cln (10 μg/mL; as a selective β2-AR agonist) and various concentrations of ICI (0.1, 0.3, and 1 μg/mL; as a selective β2-AR antagonist) for 24 h. For combinational treatment, the cells were pretreated with ICI for 45 min and then exposed to Cln for 24 h. Non-treated cells were considered the control group.

In the second step, we measured the effect of Cln on apoptosis induced by HSV-TK/GCV suicide gene therapy in the co-culture of GBM cells and OEC-TK. To determine the effective dose and time of GCV treatment on OEC-TK, cells were exposed to different concentrations of GCV (1, 2.5, 5, 10, and 20 μg/mL) for 24, 48, and 72 h. Then, the co-cultures of GBM cells and OEC-TK were treated with different concentrations of GCV for 72 h. Finally, to evaluate the Cln effect on cell viability and apoptosis, the mixed GBM cells and OEC-TK (at a ratio of 1:1) were exposed to the appropriate concentrations of GCV, Cln, and ICI for 72 h. For the combination treatment of the cells in the suicide gene therapy experiment, the cells were pretreated with ICI (0.3 μg/mL) and Cln (10 μg/mL) for 24 h according to the groups, and then the GCV was added to them.

### MTT assay

MTT assay was used to evaluate the viability of cells. The cells were cultured in 96-well cell culture plates and were exposed to the appropriate treatment according to the grouping. After the different times for each treatment, the medium containing the relevant drugs was removed from the wells, and the cells were incubated in MTT solution at a concentration of 0.5 mg/mL at 37°C for 4 h. Then, the MTT solution was eliminated and 100 μL DMSO was applied to dissolve the formazan crystals. Finally, the absorbance at 570 nm was evaluated using an ELISA reader.

### Reverse transcription-polymerase chain reaction

According to the manufacturer’s recommendations, total RNA was extracted from the cells using TRIzol reagent. The RNA concentrations were measured by evaluating A_260_/A_280_ absorption. Following quantification of RNA concentrations, cDNA was synthesized from 1 μg of total RNA using the reverse transcription kit. The expression of Cx43 and GAPDH was evaluated with real-time PCR using SYBR Premix Ex Taq. PCR was performed at 95°C for 15 min, followed by a total of 40 cycles of denaturation at 95°C for 30 s, an annealing step at 58°C for 30 s, and an extension step at 72°C for 30 s. The expression values of Cx43 were normalized to the mean of GAPDH amounts using the ΔΔCT method. The synthetic primer sequences for Cx43 and GAPDH were as follows:

Cx43 (forward: 5′-AGGTGGACTGTTTCCTCTCTC-3′, reverse: 5′-TTGCTCACTTGCTTGCTTGTTG-3′). GAPDH (forward: 5′-CTCATTTCCTGGTATGACAACGA-3′, reverse: 5′-CTTCCTCTTGTGCTCTTGCT-3′)

### Western blotting

First, total proteins were extracted from cultured cells using cold RIPA buffer, and the cell lysates were centrifuged at 13,000 × *g* for 15 min. The supernatants were collected, and the protein concentration was evaluated using the Bradford assay. Equal amounts of protein samples (30 μg per well) were separated on 12.5% SDS-PAGE and electrophoresed at 130 V for 2 h. Subsequently, the proteins were transferred onto PVDF membranes, and then, for blocking of the nonspecific areas, the membranes were incubated with 5% skimmed milk in TBST for 1 h at room temperature. The blocked membranes were incubated with the primary antibodies against Cx43 (1:2,000), HSV-TK (1:500), cleaved caspase-3 (1:500), Bcl-2 (1:500), and Bax (1:500) overnight at 4°C. Then, the membranes were washed with TBST six times for 5 min each and exposed to the HRP-conjugated secondary antibody (1:5,000) for 60 min at room temperature. After washing the membranes with TBST six times, immunoreactive bands were detected with the ECL kit. Densitometry analysis was conducted using ImageJ software. The membranes were then stripped and re-probed by primary antibody against β-actin (1:2,500) as a loading control. The amounts of each band were normalized to their β-actin levels.

### Lentiviral particle preparation

The lentiviral vector particles containing HSV-TK, GFP, and puromycin resistance genes were produced in HEK293T cells with transfecting vector plasmid pcDH513B-TK-GFP-T2A-Pur, packaging helper plasmid pPAX2, and envelope plasmid pMD2.G. The pCDH-513B-1 lentiviral vector (System Biosciences, CA, USA) containing bicistronic GFP and puromycin resistance genes under the EF1a promoter was used as control. HEK293 cells were cultured in DMEM medium supplemented by 10% FBS and 1% penicillin/streptomycin. Transfection of HEK293 cells was performed using the calcium phosphate (CaPO_4_) precipitation method. After 24, 48, and 72 h, the supernatant medium containing recombinant viral particles was collected and centrifuged at 1,500 × *g* for 10 min at 4°C to pellet the HEK293T cellular debris. The supernatant was filtered and centrifuged at 25,000 × *g* for 2 h at 4°C for final concentration.

### Transduction of OECs with lentivirus

The human OECs were transduced by the HSV-TK/GFP lentiviral vector at a multiplicity of infection of 50. The cells (at a density of 2 × 105 per well) were seeded in a 6-well culture plate and incubated at 37°C overnight. Next, the cells were exposed to the complete medium containing lentiviral supernatant for 24 h in the presence of polybrene at a concentration of 8 μg/mL. After 72 h of transduction, cells expressing the GFP gene were identified using fluorescence microscopy. Then the medium was replaced with fresh medium containing puromycin (2 μg/mL) for 72 h. The expression of HSV-TK protein was confirmed using western blot techniques.

### Flow cytometry

According to the manufacturer’s recommendations, the percentage of apoptotic cells was measured using the Annexin V/7-AAD (FITC) detection kit. The cells were cultured in a 6-well tissue plate at a density of 2 × 105 per well. After the drug treatment, the cells were harvested with trypsin and centrifuged at 2,000 × *g* for 5 min. The supernatant was removed, and the pellet was washed with cold PBS twice. Annexin V (10 μL) and 7-AAD (10 μL) solution was added to the cell suspension and incubated at room temperature for 10 min in the dark. After incubation, the cell apoptosis was analyzed using flow cytometry (BD Biosciences) evaluation.

### Statistical analysis

Statistical analysis was performed using GraphPad Prism 8 software. Data were analyzed with one-way ANOVA and two-way ANOVA followed by Tukey’s post hoc test. The data are shown as mean ± SD. The experiments were accomplished three times. p values < 0.05 were considered statistically significant.

## Data Availability

The data of this research are available from the corresponding author upon request.
